# Annotations

**Published:** 1904-02-06

**Authors:** 


### ANNOTATIONS.

### Not a Guy's but a Southwark Scandal.

The papers have again been full of a so-called "Hospital Scandal." This time the authorities of Guy's Hospital have been attacked by the Southwark Guardians for not retaining in the wards a man suffering from stricture. The facts are very simple. A month ago the man in question was brought to the hospital, where he was afforded temporary relief, and no bed being available for his reception at Guy's he was sent in a cab to St. George's Workhouse, with a recommendation for his admission into that institution. The thoughtfulness of the house surgeon in putting the man in a cab and sending him to the workhouse constitutes the "scandal" of which the Southwark Guardians complain. It thus appears that although the Guy's Hospital authorities might have done infinitely less, they could not have done more in the circumstances to protect the patient and provide for his proper treatment. Guy's treats every year 125,000 out-patients, of whom a large proportion, together with some two-fifths of the in-patients, say, 3,000, come from Southwark and Bermondsey. It has been the practice under an old regulation to require a payment of 2s. a day for every Poor-law case in the wards, but the Guardians of Southwark are, apparently, alone amongst Boards of Guardians in refusing this contribution to Guy's Hospital. The niggardliness of the Southwark Guardians would appear to be characteristic of the inhabitants of Southwark generally in the matter of hospital support, seeing that they collectively contributed £277 in 1903 towards the £90,000 expended by Guy's Hospital in that year on the relief of the sick and suffering poor. The Legislature imposes upon the Southwark Guardians the duty of providing that no necessitous person in their midst shall suffer in hunger or sickness. We are informed that the arrangements at St. George's Workhouse do not provide adequate accommodation for the reception of the sick cases which properly come within the province of the Southwark Guardians. The public will be quick to perceive therefore that this is not a hospital scandal but a Poor-law scandal, which calls for the prompt interference of the Local Government Board. That Board should compel the Southwark Guardians to immediately provide at St. George's Workhouse a reception station where sick cases, like that of the man in question, can be retained, until it is possible to remove them to the infirmary at Champion Hill. This case illustrates a danger to the sick-poor which might result from the removal of the London general hospitals to the suburbs. Had the Poor-law Infirmary for Southwark been erected on the site occupied by St. George's Workhouse, nothing would probably have been heard of the present case.

### Amusements by Death Risks.

"Hail, Cæsar, those about to die salute thee!" Such was the greeting which the Roman gladiators addressed to their emperor, as they entered the fatal arena. What would a member of the British public think if some of the brave men and women who risk their lives for his amusement should give him such a greeting as he sits in circus or music-hall to see them face death to give him a minute's, nay, half a minute's thrill? The bicycle is responsible for many of these unwholesome excitements. Some of the recent developments depend entirely for their interest to the public on the risk involved. First, there was "looping the loop," and the—to the eager public—exhilarating accidents to those who ventured the feat. But we have improved on that within eighteen months. We have had the girl who loops the loop in a motor-car. Then we had the other girl who loops the loop with a piece cut out of the bottom of the track. Last comes the man who has the piece cut out of the top of his track, and for a second's space flies through the air head downwards before he lands safe on the farther side of the gap. For a variation, we have the cyclists who do their work on the shelving sides of a bottomless basket raised high above the ground—"la cercle de la mort" as the performance was described in one playbill. On they go, knowing that a momentary pause, an involuntary swerve, may carry them from life to death. There seems no limit to the public's love of danger—other people's danger, that is. In all the cases we have mentioned there is, on the part of the spectators, something of the old Roman cruelty which could allow men and women to sit comfortably in their seats while others were risking their lives for their momentary amusement. And this is the twentieth century after Christ!

### Babies and Beauty-Doctors.

Even those who dispute the claim of Englishwomen to the palm of beauty are as a rule willing to extol the charm of the English child. But apparently the modern mother does not think her child the loveliest thing in the world, after the fond fashion of mothers of old. Else why is it solemnly recorded in a newspaper that a tiny maid of noble birth is under treatment by a popular beauty-doctor? If there is indeed any grave defect of face or figure about the child in question, surely a medical or surgical specialist is the person to consult, and surely also the fact should be kept out of the newspapers. If it is merely a matter of a complexion that is not as pink and white as may be desired, a nose that insists, baby-fashion, on turning up, or a mouth that looks too wide, it would be quite worth while to give time a chance. Plain children have before now developed into famous beauties. Mrs. Norton, whose beauty, talent, and perhaps misfortune, won the homage of all in the days when Queen Victoria was young, was the ugly duckling of, it must be admitted, an exceptionally handsome family, and on the night when she went out to make a triumphant début all that her relations said in praise was, as Fanny Kemble records in her "Reminiscences of a Girlhood," that "Caroline does not look so bad to-night." There is another aspect to the problem, for if historical instances are in any way typical, beauty does not always bring happiness to its possessor. And in any case there is a charm beyond beauty in fresh unaffected innocence, the un-selfconsciousness of youth. This is not likely to survive a course of treatment by a beauty doctor administered to a patient who is still in the nursery. The child who begins her career by being conceited about her looks or by being worried about her want of prettiness, is in either case, likely to be found wanting in that naturalness, that self-forgetfulness, that interest in things and people outside herself, which is—even from the point of view of a match-making mother—more winning than the finest complexion or the most perfect eyebrows.

